# Radiation-induced toxicities and outcomes after radiotherapy are independent of patient age in elderly salivary gland cancer patients: results from a matched-pair analysis of a rare disease

**DOI:** 10.1007/s00405-020-06393-x

**Published:** 2020-09-30

**Authors:** Alexander Rühle, Sofie Rothhaar, Erik Haehl, Tobias Kalckreuth, Tanja Sprave, Raluca Stoian, Constantinos Zamboglou, Eleni Gkika, Andreas Knopf, Anca-Ligia Grosu, Nils H. Nicolay

**Affiliations:** 1 Department of Radiation Oncology, University of Freiburg–Medical Center, Robert-Koch-Str. 3, 79106 Freiburg, Germany; 2 German Cancer Consortium (DKTK) Partner Site Freiburg, German Cancer Research Center (Dkfz), Neuenheimer Feld 280, 69120 Heidelberg, Germany; 3 Department of Otorhinolaryngology, University of Freiburg - Medical Center, Killianstr. 5, 79106 Freiburg, Germany

**Keywords:** Head-and-neck cancer, Salivary gland cancer, Radiotherapy, Chemoradiotherapy, Elderly patients

## Abstract

**Purpose:**

This study analyzed survival and toxicity after (chemo)radiotherapy for primary salivary gland cancer patients aged ≥ 65 years and compared these results with younger patients using a matched-pair analysis.

**Methods:**

Twenty-nine elderly patients with primary salivary gland carcinomas treated with (chemo)radiotherapy from 2008 to 2020 at University of Freiburg Medical Center were analyzed for oncological outcomes and therapy-associated toxicities. Local/locoregional control (LRC), progression-free survival (PFS) and overall survival (OS) were calculated using the Kaplan–Meier method, and the influence of clinical parameters on patient outcomes was assessed. A matched-pair analysis was performed after matching with patients < 65 years.

**Results:**

Nine patients (31.0%) received definitive (chemo)radiotherapy, and 20 patients (69.0%) were treated in the adjuvant setting. 2-year LRC, PFS and OS ranged at 82.4%, 53.7% and 71.8%, respectively. Smoking (HR 3.980, *p* = 0.020), reduced performance status (HR 3.735, *p* = 0.016) and higher comorbidity burden (HR 4.601, *p* = 0.005) correlated with inferior OS. Using a matched-pair analysis with younger patients, elderly patients exhibited a trend towards reduced OS (HR 3.015, *p* = 0.065), but not PFS (HR 1.474, *p* = 0.371) or LRC (HR 1.324, *p* = 0.633). Acute and chronic grade 3 toxicities occurred in 31.0% and 12.5% of elderly patients, respectively, and the matched-pair analysis revealed no significant differences between age groups regarding treatment-related toxicities.

**Conclusion:**

Treatment-related toxicities as well as LRC and PFS were comparable for salivary gland cancer patients undergoing radiotherapy. Therefore, concerns for more pronounced toxicities or reduced local/locoregional response rates should not guide treatment decisions in affected elderly patients.

**Electronic supplementary material:**

The online version of this article (10.1007/s00405-020-06393-x) contains supplementary material, which is available to authorized users.

## Introduction

Primary malignant salivary gland tumors are rare with an annual incidence of about 1 in 100,000 people, accounting for about 5% of all head-and-neck cancer cases [1, 2]. Primary salivary gland cancers form a heterogeneous group with different histological types and distinct biological behaviors [3]. The most frequent histological subtypes are mucoepidermoid and adenoid cystic carcinomas, followed by acinic cell carcinomas, adenocarcinomas, squamous cell carcinomas and salivary duct carcinomas; however the prevalence of individual histologies shifts in elderly patients [4–6]. The parotid gland constitutes the most common localization for malignant salivary gland cancers followed by minor salivary glands and the submandibular gland [7].

The median age of salivary gland cancer patients at the time of diagnosis ranges between 60 and 65 years, with about 45% of patients aged above 65 years, defined as the threshold for “elderly” patients according to the consensus definition of the United States National Institute of Aging [1, 4, 8–10]. Comorbidities are considerably more common in elderly cancer patients, complicating both surgical treatments and chemoradiotherapy in this vulnerable patient cohort [11]. For head-and-neck squamous cell carcinomas (HNSCC), it has been demonstrated that the benefit of simultaneous chemotherapy administration during radiotherapy decreases with age, but similar data are lacking for elderly patients with malignant salivary gland tumors [12–14]. As the various salivary gland cancer histologies exhibit distinct age peaks, the prognosis of elderly salivary gland cancer patients may be different to younger patients; for instance, the incidence of low-grade mucoepidermoid carcinomas, which is known to have a relatively good prognosis, is higher in younger than in elderly patients [1, 7, 9].

The treatment of malignant salivary gland tumors is challenging, as randomized trials for this entity are rare [15]. In general, complete surgical resection alone is recommended for early-stage tumors, while adjuvant radiotherapy is often applied after surgery for locally advanced tumors, nodal metastases or in case of other risk factors for recurrence such as adenoid cystic carcinoma histologies, high-grade tumors (G3/G4), close or positive resection margins, perineural or lymphovascular invasion. Patients who are medically or functionally inoperable, refuse surgery or exhibit distant metastases, can also be treated with definitive (chemo)radiotherapy, leading to 5-year local control rates of about 50% [16]. Within the context of locoregional treatment, the ideal management of the clinically negative neck (cN0) remains a controversial issue, and observation, elective neck dissection or prophylactic radiotherapy may constitute potential treatment options in dependence of histology, grading and T status [16, 17]. Similarly, the role of concomitant chemotherapy to adjuvant radiotherapy in case of incomplete resection or extracapsular lymph node extension for this entity remains unclear, and clinical workflows for salivary gland cancers often extrapolate evidence from HNSCC trials [18–20].

Here, we present the first matched-pair analysis between elderly and younger salivary gland cancer patients undergoing (chemo)therapy, aiming to examine the role of patient age regarding oncological outcomes and treatment-related toxicities.

## Material and methods

### Patients and treatment

Ethical approval for this study was given by the Ethics Committee of the University of Freiburg (Reference No. 551/18, amendment in 2020 [#200861]). All elderly patients (≥ 65 years) who received radiotherapy or chemoradiotherapy for a primary salivary gland carcinoma between 2008 and 2020 at the Department of Radiation Oncology, University of Freiburg Medical Center, were included in this analysis. Notably, patients with intraparotideal lymph node metastases from cutaneous squamous cell carcinoma or HNSCCs were not included in our analysis. Demographic characteristics and clinical data were retrospectively assessed from the electronic patient records. Histopathology data during treatment were extracted from the internal pathology reports. A positive smoking status was considered for patients with a tobacco consumption of at least 10 pack years. The comorbidity burden of salivary gland cancer patients was quantified using the validated age-adjusted Charlson comorbidity index (CCI) [21]. Staging of salivary gland tumors was based on the 7th Edition of the UICC TNM classification.

**Fig. 1:**
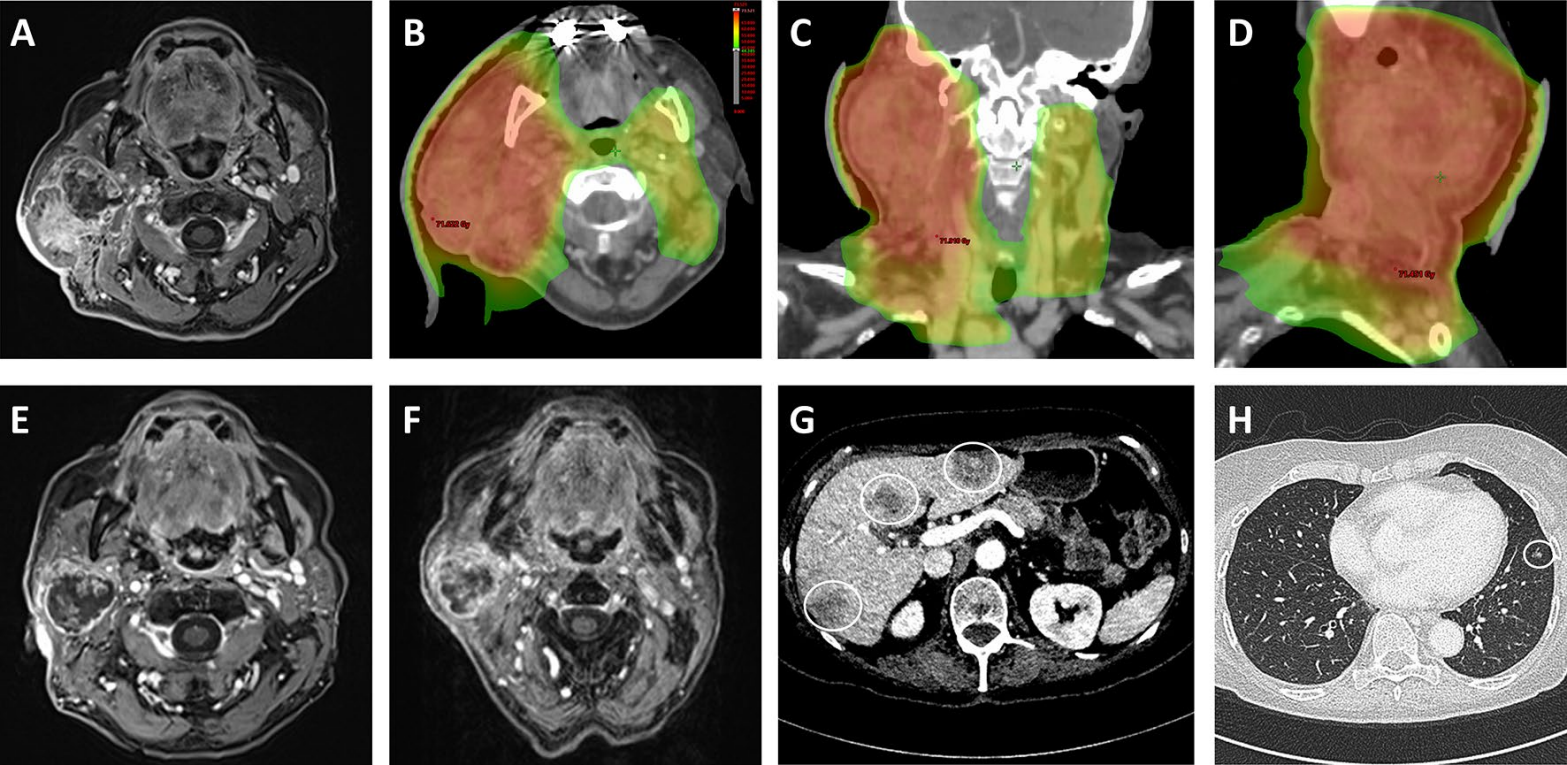
Definitive chemoradiotherapy for an undifferentiated (G4) parotid gland carcinoma in a 76-year-old patient. The locally advanced parotid gland carcinoma (cT4a cN2b cM0) was treated with intensity-modulated radiotherapy in a simultaneous-integrated boost-concept (SIB) between December 2019 and January 2020. While the high-risk PTV received 69.3 Gy delivered in 33 fractions, the medium-risk PTV and low-risk PTV were treated with 62.7 Gy and 56.1 Gy, respectively. **a** Pre-therapeutic contrast-enhanced T1-weighted MRI image in October 2019 demonstrating a right-sided parotid gland carcinoma infiltrating the cutis and the sternocleidomastoid muscle with ipsilateral lymph node metastases in level II, III, IV and V. **b–d** Radiotherapy treatment plan showing the dose distribution in an axial (**b**), coronary (**c**) and sagittal (**d**) view. **e**, **f** Follow-up images in March 2020 (**e**) and June 2020 (**f**) showing regression of the primary tumor and lymph node metastases with no signs for local or locoregional recurrence. **g, h** However, multiple hepatic hypodensities (**g**) and pulmonary noduli (**h**) suspicious for distant progression occurred in June 2020

Treatment decisions were based on the recommendations of the multidisciplinary tumor board. In general, definitive radiotherapy was only applied in patients who refused surgery or were medically or technically inoperable, while adjuvant radiotherapy was recommended for locally advanced (T3/T4) tumors, positive nodal status or other risk factors including tumor histology (adenoid cystic carcinoma, high-grade tumors), resection margin (close or positive margin) or perineural/lymphovascular invasion. Extrapolating the results of the HNSCC landmark trials EORTC 22931 and RTOG 9501, postoperative chemoradiotherapy was scheduled for patients with incomplete resection or extranodal extension, unless there were medical contraindications against chemotherapy usage [18–20]. For radiotherapy planning and treatment, patients were immobilized using individually molded thermoplastic masks. Radiotherapy planning was carried out utilizing Oncentra MasterPlan® (Nucletron BV, Veenendaal, The Netherlands) and Eclipse™ planning software (Varian Medical Systems)., Three-dimensional conformal radiotherapy or intensity-modulated radiotherapy were used for radiotherapy dependent on the time period of treatment. In general, follow-up care of treated patients consisted of 3-monthly intervals in the first year after treatment, followed by 6-month intervals between years 2 and 5. In case of clinical progression or severe treatment-related toxicities, patients presented in shorter intervals. As patients treated between 2008 and 2020 were included in our study, not all patients did pass the complete follow-up care of 5 years. Follow-up consultations consisted of a physical examination and cross-sectional imaging of the head-and-neck region using CT or MR imaging (Fig. 1).

### Survival analysis

Patients who were lost to follow-up were censored at the time of last contact for the following analyses, and the reverse Kaplan–Meier method was used to calculate the median follow-up. Overall survival (OS) was defined as the interval from radiotherapy initiation to death from any cause, while progression-free survival (PFS) was calculated as the time between start of treatment to disease progression or death from any cause. For the calculation of local/locoregional control (LRC), the time from treatment initiation to the absence of any progression of the primary tumor or occurrence of cervical lymph node metastases was assessed.

### Toxicity analysis

Both acute and chronic treatment-related toxicities were classified according to the Common Terminology Criteria of Adverse Effects (CTCAE) version 5.0. All toxicities that occurred during radiotherapy treatment and within the first 90 days after completion, were classified as acute toxicities.

### Statistical analysis

LRC, PFS and OS were determined according to the Kaplan–Meier method, and log-rank tests were used to reveal potential differences in survival. Univariate Cox proportional hazards model analyses were carried out in order to study the influence of clinical and pathological parameters. All primary salivary gland cancer patients < 65 years that received (chemo)radiotherapy treatment between 2008 and 2020 at our institution were used for case–control matching with the elderly population. The database search revealed 28 primary salivary gland cancer patients who were younger than 65 years at the time of radiotherapy treatment. A case–control matching was performed using the prognostic parameters smoking, performance status (ECOG 0 vs. 1–2) and comorbidity burden (CCI 2 versus 3–8) as matching variables. The tolerance level for these three categorial variables was set at 0, leading to a matched-pair dataset with 20 patients per age group. Chi-square tests for ordinal variables and unpaired *t* tests for interval variables were used to test for differences between both groups. A *p* value below 0.05 was considered significant for all analyses. Statistical analyses including case–control matching were conducted using IBM SPSS Statistics software version 25 (IBM, Armonk, NY, USA).

## Results

### Demographic characteristics

A total of 29 elderly patients with histologically confirmed primary salivary gland carcinomas underwent radiotherapy in our department between 2008 and 2020 (Table 1). 17 patients (58.6%) were female and 12 patients were male (41.4%). The median age was 75 years with an age span ranging between 66 and 89 years. Only six patients (20.7%) had a positive smoking history. Nineteen patients (65.5%) exhibited an ECOG performance status of 0, and seven patients (24.1%) had an ECOG status of 1, indicating a relatively fit elderly patient group at the time of radiotherapy. The CCI was used to quantify the patients’ comorbidity burden and ranged between 2 and 8 with a median CCI value of 2, showing that most elderly patients did not exhibit relevant comorbidities. Most tumors were locally/locoregionally advanced, indicated by a high proportion of T3/T4 tumors (*n* = 17, 58.6%) or nodal metastases (*n* = 17, 58.6%). The most common histologies were adenocarcinomas (*n* = 12, 41.4%) and the remaining patients suffered from a plethora of different histologies [adenoid cystic carcinoma (*n* = 3), squamous cell carcinoma (*n* = 2), mucoepidermoid carcinoma (*n* = 2), acinic cell carcinoma (*n* = 2), salivary duct carcinoma (*n* = 1), carcinoma ex pleomorphic adenoma (*n* = 1), etc.; for details see Table 1]. Cancers were most commonly located within the parotid gland (*n* = 24, 82.8%).

**Table 1:** Patient characteristics consisting elderly salivary gland cancer patients treated by (chemo)radiotherapy between 2008 and 2020 (*n* = 29)

	Median (range)	
	*n*	%
Age	75 (66–89)	
Sex		
Male	12	41.4
Female	17	58.6
Smoking		
Non-smoker	23	79.3
Smoker	6	20.7
ECOG		
0	19	65.5
1	7	24.1
2	3	10.3
CCI		
2	20	69.0
3–8	9	31.0
T stage		
T1	5	17.2
T2	7	24.1
T3	8	27.6
T4	9	31.0
N stage		
N0	12	41.4
N1	7	24.1
N2	8	27.6
N3	2	6.9
M stage		
M0	29	100.0
M1	0	0.0
Histology		
Adenocarcinoma	12	41.4
Adenoid cystic carcinoma	3	10.3
Squamous cell carcinoma	2	6.9
Mucoepidermoid carcinoma	2	6.9
Acinic cell carcinoma	2	6.9
Salivary duct carcinoma	1	3.4
Carcinoma in pleomorphic adenoma	1	3.4
Others^a^	5	17.2
Unknown	1	3.4
Localization		
Parotid gland	24	82.8
Submandibular gland	2	6.9
Minor salivary glands	3	10.3
Grading		
G1	1	3.4
G2	12	41.4
G3	11	37.9
G4	2	6.9
Unknown	3	10.3

^a^Undifferentiated carcinoma (*n* = 2), lymphoepithelial carcinoma (*n* = 1), myoepithelial carcinoma (*n* = 1), large cell carcinoma (*n* = 1)

More than two-thirds (*n* = 20, 69.0%) of the patients were treated with adjuvant (chemo)radiotherapy after surgery and received a median cumulative dose of 66.0 Gy (range 59.4–70.0 Gy) (Table 2). For patients undergoing definitive (chemo)radiotherapy (*n* = 9, 31.0%), the median radiation dose ranged at 69.3 Gy (range 30.0–70.0 Gy). The median fraction doses for both adjuvant and definitive treatments were 2.0 Gy.

**Table 2:** Treatment details for (chemo)radiotherapy of elderly salivary gland cancer patients (*n* = 29)

Radiation therapy	*n*	%
Definitive	9	31.0
Adjuvant	20	69.0
Completed	26	89.7
Definitive		
Median primary tumor radiation dose	69.3 Gy	
Median elective nodal radiation dose	52.0 Gy	
Median primary tumor single dose	2.0 Gy	
Adjuvant		
Median primary tumor radiation dose	66.0 Gy	
Median elective nodal radiation dose	50.0 Gy	
Median primary tumor single dose	2.0 Gy	

Concomitant chemotherapy	*n*	%
Planned	9	31.0
Completed	6	66.7

A total of 26 patients (89.7%) completed their course of radiotherapy, but only 6 of 9 patients, who were scheduled for concomitant chemotherapy, completed their chemotherapy treatment (66.7%).

### Survival analyses

**Fig. 2:**
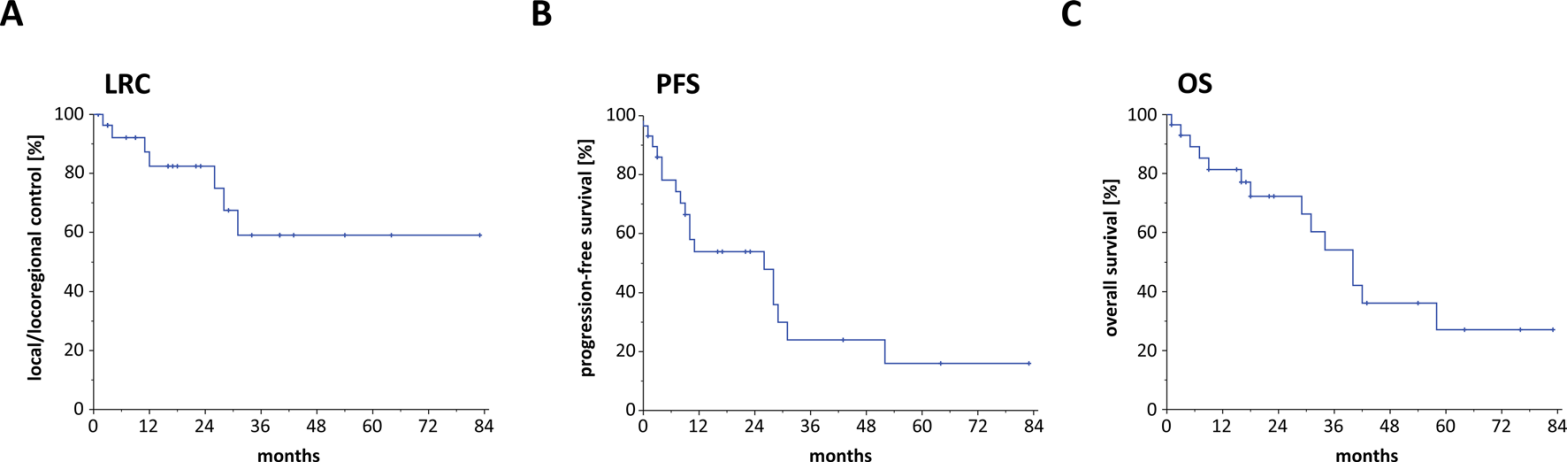
Kaplan–Meier curves showing LRC (**a**), PFS (**b**) and OS (**c**) of elderly salivary gland cancer patients treated between 2008 and 2020

After a median follow-up of 43 months, the median PFS amounted to 26 months and the median OS to 40 months. 2-year LRC, PFS and OS ranged at 84.1%, 53.2% and 69.6%, respectively (Fig. 2). Based on the consensus definition of the United States National Institute of Aging, that distinguishes between “young old” (65–74 years) and “older old” patients (≥ 75 years), we analyzed the age dependence of patient outcomes. In our analysis, “young old” patients did not exhibit superior OS rates [HR (reference: 65–74 years) = 0.653, 95% CI 0.226–1.884, *p* = 0.430] compared to patients aged 75 years or older (Table 3).

**Table 3:** Univariate Cox analysis of clinical and pathological parameters regarding OS in elderly salivary gland cancer patients receiving radiotherapy (*n* = 29)

Parameter	PFS			OS		
	HR	CI 95%	*p* value	HR	CI 95%	*p* value
≥ 75 / 65–74 years	0.720	0.285–1.821	0.488	0.653	0.226–1.884	0.430
Male/female	2.272	0.865–5.973	0.096	2.079	0.717–6.025	0.178
Smoker/non-smoker	2.281	0.789–6.596	0.128	3.980	1.243–12.748	**0.020**
ECOG 1–2/0	7.723	2.347–25.411	**0.001**	3.735	1.280–10.901	**0.016**
CCI 3–8/2	6.393	2.046–19.980	**0.001**	4.601	1.578–13.414	**0.005**
T3–T4/T1–T2	3.530	1.155–10.788	**0.027**	2.769	0.857–8.944	0.089
N1–N3/N0	1.847	0.686–4.972	0.225	3.050	0.936–9.945	0.064
G3–G4/G1–G2	2.302	0.747–7.096	0.146	1.405	0.426–4.630	0.576
Definitive/adjuvant	1.240	0.434–3.543	0.688	1.484	0.452–4.868	0.515
No chemotherapy/chemotherapy	1.797	0.626–5.157	0.276	1.532	0.477–4.925	0.474

*p* values in bold display significant results

**Fig. 3:**
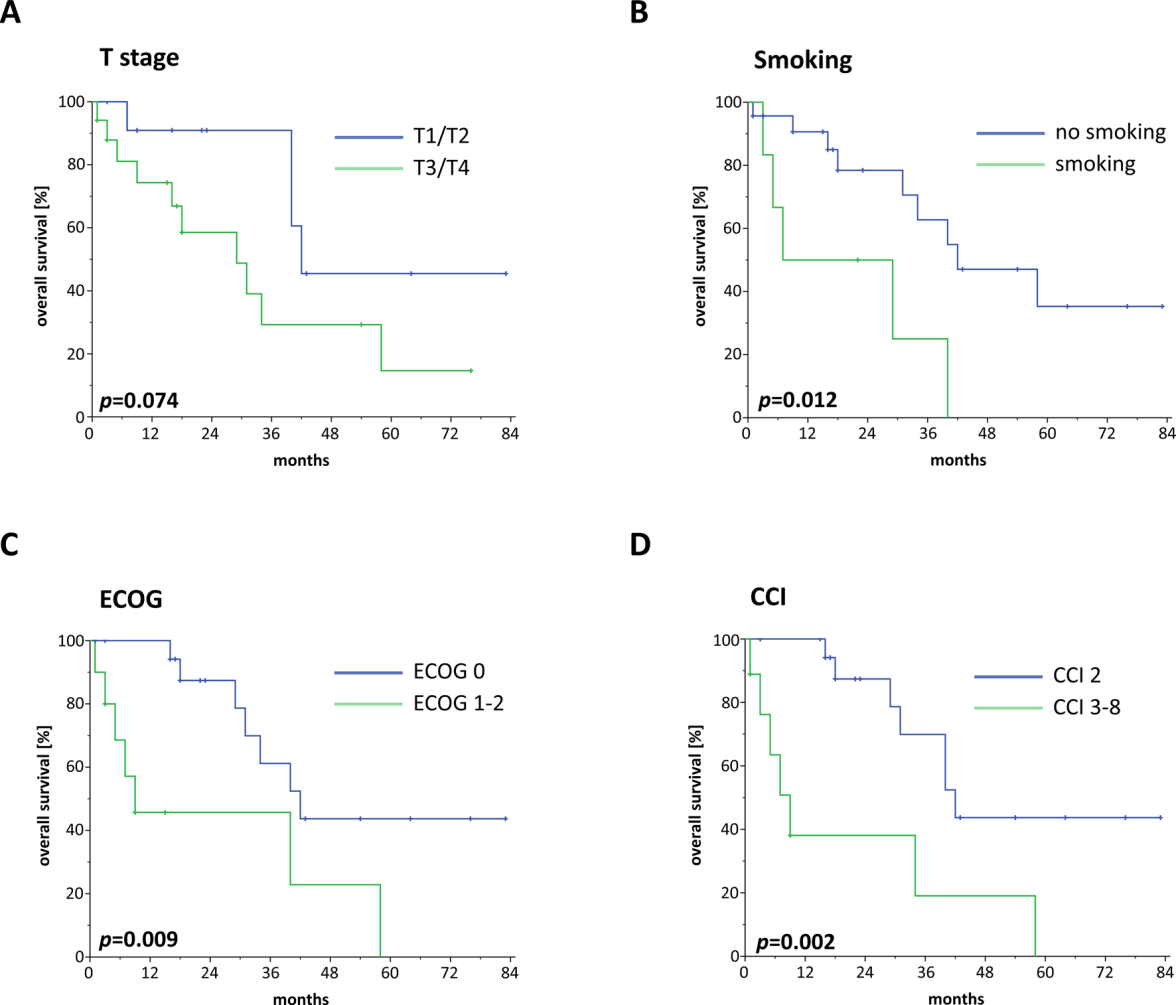
Kaplan–Meier OS curves in dependence of T stage (**a**), smoking status (**b**), ECOG performance status (**c**) and CCI (**d**). *P* values of log-rank tests are shown

Increased T stages resulted in significantly reduced PFS (HR 3.530, 95% CI 1.155–10.788, *p* = 0.027) and a trend towards impaired OS (HR 2.769, 95% CI 0.857–8.944, *p* = 0.089) in elderly salivary gland cancer patients (Fig. 3a). In contrast, the presence of cervical lymphonodal metastases was not associated with reduced PFS (HR 1.847, 95% CI 0.686–4.972, *p* = 0.225), although a trend towards reduced OS (HR 3.050, 95% CI 0.936–9.945, *p* = 0.064) was observed. Grading did not influence PFS (HR 2.302, 95% CI 0.747–7.096, *p* = 0.146) or OS (HR 1.405, 95% CI 0.426–4.630, *p* = 0.576) in our cohort.

Similarly to elderly HNSCC patients, salivary gland cancer patients with a positive smoking status were demonstrated to have significantly impaired OS rates (HR 3.980, 95% CI 1.243–12.748, *p* = 0.020), while the PFS (HR 2.281, 95% CI 0.789–6.596, *p* = 0.128) and LRC (HR 0.783, 95% CI 0.094–6.535, *p* = 0.821) did not differ between smokers and non-smokers (Fig. 3b). A considerable influence of the patients’ performance status regarding oncological outcomes could be observed in our patient cohort: while patients with an ECOG performance status of 0 had a median OS of 42 months, the median OS of patients with an ECOG status of 1 or 2 ranged at only 9 months (HR 3.735, 95% CI 1.280–10.901, *p* = 0.016) (Fig. 3c). As the median CCI amounted to 2 in our cohort, we used this value as a cut-off for the Cox regression analysis: Elderly salivary gland cancer patients exhibiting a CCI of 3 or higher (*n* = 9, 31.0%) were shown to have considerably deteriorated PFS (HR 6.393, 95% CI 2.046–19.980, *p* = 0.001) and OS (HR 4.601, 95% CI 1.578–13.414, *p* = 0.005) in comparison with patients that exhibited a CCI of 2 and therefore no significant comorbidities (Fig. 3d). Neither LRC (HR 3.321, 95% CI 0.721–15.305, *p* = 0.124) nor PFS (HR 1.240, 95% CI 0.434–3.543, *p* = 0.688) nor OS (HR 1.484, 95% CI 0.452–4.868, *p* = 0.515) were significantly different for patients receiving definitive radiotherapy compared to patients receiving adjuvant radiotherapy after surgery, and concomitant chemotherapy as applied in 9 patients (31.0%) did not lead to superior PFS (HR 0.556, 95% CI 0.194–1.597, *p* = 0.276) or OS (HR 0.653, 95% CI 0.203–2.098, *p* = 0.474).

### Matched-pair analysis

A total of 28 primary salivary gland cancer patients between 28 and 63 years who were treated with (chemo)radiotherapy were identified. As the smoking status, performance status and comorbidity burden were demonstrated to be prognostic parameters in our elderly patient cohort, these factors were used as variables for case–control matching. The tolerance factor of these 3 matching variables was set to 0, leading to exact matches regarding these variables. Twenty matching pairs were found and compared regarding clinical, pathological and treatment-related parameters using Chi-square tests and t tests (supplementary Table 1).

The median age in the elderly cohort amounted to 74 years (range 66–89 years), while it was 47.5 years (range 28–63 years) in the matched cohort (*p* < 0.001, unpaired *t* test). While there were no significant differences regarding the TNM parameters, the submandibular gland and minor salivary glands were more frequently affected in younger patients, although statistical significance was not reached (*p* = 0.060, Chi-square test). There was a trend towards a higher prevalence of poorly or undifferentiated tumors in elderly salivary gland cancer patients in comparison with their matching younger counterparts (*p* = 0.054). Histology distribution significantly differed between both groups (*p* = 0.048): while adenocarcinoma (*n* = 11) was the most common histology in the elderly cohort, adenoid cystic carcinomas (*n* = 7) and mucoepidermoid carcinomas (*n* = 5) were most frequent in the matched cohort consisting of younger patients. One quarter (*n* = 5, 25%) received definitive (chemo)radiotherapy in the elderly cohort, which was considerably higher than in the control group of younger patients (*n* = 1, 5.0%; *p* = 0.077). There were no differences concerning administration of concomitant chemotherapy between elderly and younger patients (*p* = 0.168).

**Fig. 4:**
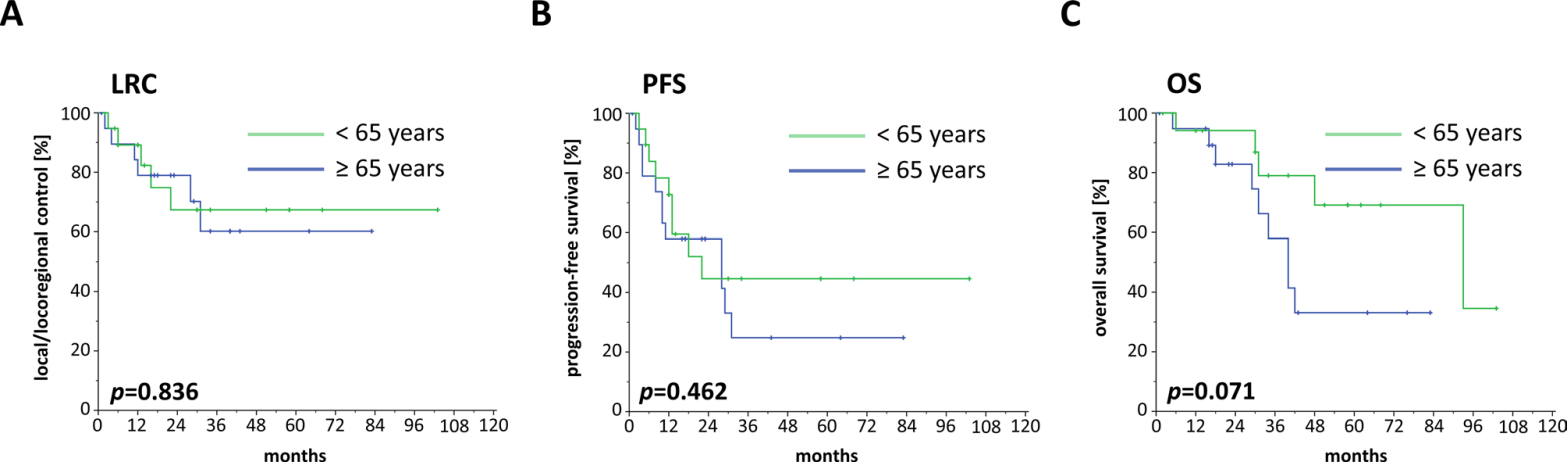
LRC (**a**), PFS (**b**) and OS (**c**) of salivary gland cancer patients receiving (chemo)radiotherapy stratified by the patient’s age. Elderly patients were matched pairwise for the prognostic parameters smoking, performance status and comorbidity burden quantified by the CCI. *P* values of log-rank tests are shown

LRC (HR 1.134, 95% CI 0.345–3.720, *p* = 0.836) and PFS (HR 1.376, 95% CI 0.579–3.272, *p* = 0.470) did not differ between young and elderly primary salivary gland cancer patients after matching, but elderly patients exhibited a trend towards reduced OS (HR 2.835, 95% CI 0.863–9.312, *p* = 0.086). Median OS ranged at 93 months for the younger cohort, which was more than twice as long than for the elderly group (median 40 months) (*p* = 0.071, log-rank test) (Fig. 4). Considering that neither prognostic clinical parameters (smoking, performance status, CCI) nor TNM stages differed between both groups, it can be concluded that the lower OS of elderly salivary gland cancer patients receiving radiotherapy was not related to these factors.

### Toxicity

The proportion of patients suffering from high-grade acute toxicities was found to be moderate with 31.0% of patients (*n* = 9) exhibiting acute CTCAE grade 3 toxicities (supplementary Table 2). The most common acute grade 3 toxicity was mucositis (*n* = 5) followed by dermatitis (*n* = 2) and cytopenia (*n* = 2) (supplementary Table 3). Notably, no acute grade 4 or 5 toxicity occurred in our cohort.

Chronic toxicities could be assessed in 24 patients, of which 20 patients (83.3%) exhibited CTCAE grade 1/2 toxicities. Only 3 patients (12.5%) suffered from chronic CTCAE grade 3 toxicities in our dataset, one with chronic pain symptoms requiring continuous analgesic medication due to an osteoradionecrosis, another with mucositis and thrush receiving antifungal medication and another with chronic lymph edema and severe pain.

The toxicity analyses of the matched-pair cohort revealed a similar toxicity pattern between younger and elderly patients (Table 4). Among the 20 elderly salivary gland cancer patients in the matched-pair analysis, about two-quarters (*n* = 14, 70.0%) suffered from mild (CTCAE grade 1/2) acute toxicities, while 6 patients (30.0%) exhibited CTCAE grade 3 acute adverse effects. In comparison, 12 young patients (60.0%) had CTCAE grade 1/2 acute adverse reactions and 8 patients (40.0%) CTCAE grade 3/4 acute toxicities (*p* = 0.540, Chi-square test). Similar to the acute toxicities, chronic toxicities did not significantly differ between both age groups in the matched-pair analysis (*p* = 0.071): while the prevalence of chronic grade 2 toxicities (especially xerostomia) was higher in younger patients (*n* = 9, 45.0%) than in the elderly cohort (*n* = 3, 15.8%), there were 3 chronic grade 3 adverse reactions (see above) in elderly patients, whereas no chronic grade 3 toxicity was found in the matching younger patients.

**Table 4:** Toxicity results of elderly patients compared to matching younger patients with salivary gland cancer

Acute	Elderly cohort (≥ 65 years)		Matched cohort (18–65 years)		*p* value
	*n*	%	*n*	%	
CTCAE 1/2	14	70.0	12	60.0	
CTCAE 3	6	30.0	7	35.0	
CTCAE 4	0	0.0	1	5.0	
CTCAE 5	0	0.0	0	0.0	0.540

Chronic	*n* = 19		*n* = 20		
CTCAE 0	1	5.3	0	0.0	
CTCAE 1	12	63.2	11	55.0	
CTCAE 2	3	15.8	9	45.0	
CTCAE 3	3	15.8	0	0.0	
CTCAE 4/5	0	0.0	0	0.0	0.071

## Discussion

Our results derived from a retrospective single-center matched-pair analysis show that elderly salivary gland patients who were treated with (chemo)radiotherapy exhibited respectable LRC rates, but relatively poor survival rates, probably reflecting the advanced age of our treatment cohort. We could identify smoking, performance status and comorbidity burden as prognostic parameters for elderly patients with primary salivary gland cancer undergoing radiotherapy. To the best of our knowledge, this is the first matched-pair analysis comparing the oncological results and toxicity patterns of elderly salivary gland cancer patients undergoing (chemo)radiotherapy with their younger counterparts. The matched-pair analysis with younger salivary gland patients, in which smoking, ECOG status und CCI were used as matching variables, revealed comparable LRC and PFS but a trend towards worsened OS in the elderly cohort, probably caused by more non-cancer related deaths in this group. For instance, previous meta-analyses comprising HNSCC patients have reported about non-cancer related death in up to 40% of patients aged 70 years and older [22]. (Chemo)radiotherapy-related toxicities were moderate in our elderly cohort with a grade 3 toxicity prevalence of 31.0% (acute) and 12.5% (chronic) without any grade 4 or grade 5 adverse reactions. Considering the high completion rate of almost 90% and the fact, that we did not observe significant differences in the toxicity profile and severity between younger and elderly patients, our results suggest that radiotherapy is a feasible and relatively well-tolerated treatment modality also for elderly patients with primary salivary gland cancer.

Over the last decades, treatment approaches for high-grade and/or locally advanced salivary gland cancer have changed from surgery alone to a multimodal therapy consisting of primary resection and adjuvant (chemo)radiotherapy [23]. Multivariate analyses of the SEER database with more than 2000 patients revealed significantly superior survival rates for high-grade and for locally advanced salivary gland cancer patients who received adjuvant radiotherapy [24]. Definitive (chemo)radiotherapy is considered to be a treatment alternative for inoperable patients or patients with distant metastases.

Contrary to HNSCC, for which smoking is known to be a key risk factor for tumor development, the role of smoking is of less importance in the pathogenesis of salivary gland cancers, which is reflected in our data with only about 20% of patients being smokers [25]. Interestingly, smoking was identified as a significant prognostic parameter for OS but not for PFS in our analysis. In another analysis comprising 24 submandibular gland carcinoma patients, distant metastasis-free survival was significantly lower for smokers than for non-smokers [26]. Guidelines for head-and-neck cancers recommend to inform patients about the risk of continuing smoking and about the potential benefits regarding smoking cessation, as smoking may lower the efficacy of radiotherapy and increase the risk for second primary tumors and cardiovascular diseases [27].

The distribution of salivary gland tumor histologies shifts with increasing age at the time of diagnosis. Mucoepidermoid carcinomas have a generally good prognosis compared to other salivary gland histologies, but these tumors have been demonstrated to be considerably less prevalent in elderly salivary gland cancer patients [4, 28]. In turn, the prevalence of adenoid cystic carcinoma, that exhibits a rather poor prognosis, is considerably higher in younger patients and may abrogate this effect.

Especially for elderly cancer patients, the comorbidity burden quantified by the CCI or ACE-27 score is known to have a prognostic value [29]. In contrast to elderly HNSCC patients, the comorbidity burden in salivary gland cancer patients is commonly lower and comparable with other malignancies not related to smoking or alcohol consumption such as prostate cancer [30]. In a large retrospective study comprising 666 salivary gland cancers, the ACE-27 score was found to significantly correlate with OS, whereas the disease-specific survival was not affected [30]. In our study, both ECOG performance status and CCI were able to discriminate the survival rates of elderly patients with salivary gland cancer after (chemo)radiotherapy; however, a standardized survey of the patients’ comorbidities using the CCI or ACE-27 may reduce the intra- and interobserver variability that is known to be an issue regarding the assessment of the performance status [31].

The role of systemic treatment for salivary gland cancer patients is controversial, as larger prospective trials investigating this aspect are lacking [32–35]. In our small dataset, chemotherapy administration did not influence PFS or OS, although this should be interpreted cautiously, as the sample size was small and chemotherapy administration was applied for patients exhibiting risk factor for tumor recurrence.

In a large analysis based on the Danish salivary gland cancer database including 871 patients, increased age (≥ 70 years) was demonstrated to be associated with an impaired performance status as well as a higher prevalence of high-grade and advanced tumors compared to younger patients (< 70 years) [28]. Interestingly, the multivariate analysis did not reveal a significant prognostic role of increased age, whereas performance status, histological high-grade subtypes and UICC stages III/IV remained significant prognosticators for disease-specific survival. Furthermore, the authors observed an increased usage of definitive radiotherapy (10% versus 6%) in elderly salivary gland cancer patients. Similar to the large Danish database analysis, we found a trend towards a higher prevalence of higher-graded tumors (G3/G4) in elderly patients. In another retrospective study including 62 patients with mucoepidermoid carcinoma who received either definitive or adjuvant radiotherapy, increased age (above 56 years) was the only significant parameter for diminished LRC and OS [36]. O’Brien et al. could show in a retrospective study consisting of 113 patients with salivary gland cancers that age remained a significant prognostic factor even in the multivariate analysis [37].

We could demonstrate in the matched-pair analysis that treatment-related toxicities were at a moderate level and did not differ between age groups, suggesting that age-dependent treatment modifications are of minor importance for radiotherapy of salivary gland cancers. This may be due to the smaller treatment volumes and preservation of critical structures such as the contralateral salivary glands or the oral/pharyngeal mucosa. In a retrospective analysis studying the results of elderly HNSCC patients receiving radiotherapy, the authors observed comparable toxicity rates between patients < 65 years and patients aged 65 years or older [38]. In contrast, a large analysis of the Radiation Therapy Oncology Group (RTOG) including the toxicity results of 3 RTOG trials for locally advanced HNSCC (RTOG 9111, 9703, and 9914) reported about a significantly higher prevalence of high-grade toxicities with older age [39]. In another analysis consisting of 98 head-and-neck cancer patients aged 80–92 years who received radiotherapy between 1991 and 1995, only 3 patients (3.1%) developed severe late toxicities after radiotherapy, showing that even with older radiotherapy techniques, the toxicity profile was moderate in the “very old/oldest old” population [40].

Particle irradiation such as neutron or carbon ion irradiation offers some physical and biological advantages compared to photon radiotherapy. For instance, particle ions deliver most ionization energy at a well-defined depth (so-called Bragg peak) with a sharp dose fall thereafter and induce more complex DNA lesions, leading to a higher biological effectiveness. Especially adenoid cystic carcinoma, that is traditionally considered as a radioresistant tumor, may benefit from particle irradiation due to the possibility for dose escalation due to the steep dose gradient of particle irradiation. Several reports showed encouraging LRC rates for adenoid cystic carcinoma using particle irradiation [41–43], and in a small randomized trial of the RTOG and Medical Research Council (MRC) including 32 patients with inoperable, recurrent or unresectable salivary gland cancer, neutron irradiation resulted in superior LRC and a trend towards increased OS (2-year OS of 62% for neutrons versus 25% for photons, *p* = 0.01) compared to conventional photon irradiation [44]. However, larger randomized trials are necessary to define the value of particle irradiation in comparison with modern photon irradiation techniques such as intensity-modulated radiation therapy or stereotactic radiotherapy for malignant salivary gland tumors.

Despite being the first study focusing on elderly salivary gland cancer patients undergoing radiotherapy with an integrated matched-pair analysis to elucidate the role of patients’ age on survival, our study exhibits several limitations mainly due to its retrospective character. Although the parameters smoking, performance status and TNM stage were equally distributed between both age groups in the matched-pair analysis, salivary gland histologies and resulting prognoses differed depending on age. Furthermore, the small sample size did not allow for multivariate regression analyses, pointing out the importance of multicentric analyses of elderly salivary gland cancer patients given the rarity of this disease. Although physician-assessed toxicities did not differ between elderly and young patients, patient-reported outcomes and quality of life may be different between both age groups, and we were not able to retrospectively assess these parameters in our cohort. Since this is a retrospective study, especially comorbidities and treatment-related toxicities were difficult to survey in some cases. However, retrospective assessment of comorbidities using medical charts, anesthetic sheets and referral letters of extern physicians has been shown to be a reliable method [45].

In summary, elderly salivary gland cancer patients receiving radiotherapy exhibit respectable LRC rates, but relatively poor survival rates. The comparable LRC and PFS between younger and elderly salivary gland cancer patients after matching for prognostic parameters show that the probability for curation is not impaired in advanced ages; however, there was a trend towards reduced OS in elderly patients, which may be caused by more non-cancer related deaths. Due to the prognostic value of smoking, cessation of smoking should be encouraged by otorhinolaryngology surgeons, radiation oncologists and medical oncologists. The low-to-moderate prevalence of high-grade toxicities, which was comparable between young and elderly patients in the matched-pair analysis, suggests that the current standard treatment can be safely applied and that curative treatment with sufficient irradiation dose and irradiation volumes should not be omitted for elderly salivary gland cancer patients.

## Electronic supplementary material

Below is the link to the electronic supplementary material.

Supplementary file1 (PDF 168 kb)

## Data Availability

The datasets used and analyzed during the current study are available from the corresponding author on reasonable request.
