# Supplementary material for: Radiation-induced toxicities and outcomes after radiotherapy are independent of patient age in elderly salivary gland cancer patients: results from a matched-pair analysis of a rare disease
Source: Eur Arch Otorhinolaryngol. 2020 Sep 30;278(7):2537–48. doi: 10.1007/s00405-020-06393-x (PMC8165074; doi:10.1007/s00405-020-06393-x)
Supplement: Supplementary file 1 — Supplementary file1 (PDF 168 kb) [file 405_2020_6393_MOESM1_ESM.pdf]

Supplementary table 1: Distribution of patient characteristics after pair-matching (n=20 for each group).

		**Elderly cohort (≥65 years)**		**Matched cohort (18-65 years)**		***p*-value**
		**Median (range)**		**Median (range)**		
**Age**		74 (66 – 89)		47.5 (28 – 63)		**<0.001**
		**n**	**%**	**n**	**%**	
**Sex**	male	8	40.0	9	45.0	0.749
	female	12	60.0	11	55.0	
**Smoking**	non-smoker	16	80.0	16	80.0	1.000
	smoker	4	20.0	4	20.0	
**ECOG**	0	17	85.0	17	85.0	1.000
	1-2	3	15.0	3	15.0	
**CCI**	2	17	85.0	17	85.0	1.000
	3-8	3	15.0	3	15.0	
**T stage**	T1/T2	9	45.0	13	65.0	0.204
	T3/T4	11	55.0	7	35.0	
**N stage**	N0	10	50.0	11	55.0	0.752
	N1/N2/N3	10	50.0	9	45.0	
**M stage**	M0	20	100.0	19	95.0	0.311
	M1	0	0.0	1	5.0	
**Localization**	parotid gland	17	85.0	10	50.0	0.060
	submandibular gland	2	10.0	6	30.0	
	minor salivary glands	1	5.0	4	20.0	
**Grading**	G1/G2	8	40.0	15	75.0	0.054
	G3/G4	10	50.0	5	25.0	
	unknown	2	10.0	0	0.0	
**Histology**	adenocarcinoma	11	55.0	3	15.0	**0.048**
	squamous cell carcinoma	2	10.0	1	5.0	
	mucoepidermoid carcinoma	1	5.0	5	25.0	
	acinic cell carcinoma	2	10.0	2	10.0	
	adenoid cystic carcinoma	1	5.0	7	35.0	
	carcinoma in pleomorphic adenoma	0	0.0	1	5.0	
	others	3[a]	15.0	1[b]	5.0	
**Concept**	definitive	5	25.0	1	5.0	0.077
	adjuvant	15	75.0	19	95.0	
**Chemotherapy**	no chemotherapy	12	59.1	16	81.8	0.168
	chemotherapy	8	40.9	4	19.2	

[a]undifferentiated carcinoma (n=1), lymphoepithelial carcinoma (n=1), myoepithelial carcinoma (n=1)
[b]liposarcoma

Supplementary table 2: Toxicity results after radiotherapy or chemoradiotherapy of elderly patients with salivary gland cancer according to the CTCAE v5.0.

**Acute (n=29)**	**n**	**%**
CTCAE 1/2	20	69.0
CTCAE 3	9	31.0
CTCAE 4/5	0	0.0
**Chronic (n=24)**		
CTCAE 0	1	4.2
CTCAE 1 – 2	20	83.3
CTCAE 3	3	12.5
CTCAE 4/5	0	0.0

Supplementary Table 3: Toxicity results consisting various (chemo)radiotherapy-related adverse reactions according to the Common Terminology Criteria for Adverse Events (CTCAE) v5.0.

	**CTCAE grade**					
**Acute**	**0**	**1**	**2**	**3**	**4**	**5**
dermatitis	1	10	16	2	0	0
dysphagia	16	9	3	1	0	0
nausea	28	1	0	0	0	0
mucositis	8	4	12	5	0	0
xerostomia	16	12	1	0	0	0
dysgeusia	16	9	4	0	0	0
pain	14	7	8	0	0	0
cytopenia[a]	14	9	3	2	0	0
weight loss[b]	10	2	5	0	0	0
acute kidney injury[c]	19	4	1	0	0	0
**Chronic (n=24)**						
skin toxicity	13	8	2	1	0	0
dysphagia	17	5	2	0	0	0
mucositis	22	0	1	1	0	0
xerostomia	3	19	2	0	0	0
dysgeusia	9	13	2	0	0	0
pain	18	4	0	2	0	0
cytopenia[d]	18	2	1	0	0	0

[a]unknown in 1 case
[b]unknown in 12 cases
[c]missing in 5 cases
[d]missing in 3 cases
